# Etiology of Idiopathic Macular Holes in the Light of Estrogen Hormone

**DOI:** 10.3390/cimb45080400

**Published:** 2023-07-29

**Authors:** Nousal Wergenthaler, H. Burkhard Dick, Teresa Tsai, Stephanie C. Joachim

**Affiliations:** Experimental Eye Research Institute, University Eye Hospital, Ruhr-University Bochum, In der Schornau 23-25, 44892 Bochum, Germany; burkhard.dick@kk-bochum.de (H.B.D.); teresa.tsai@rub.de (T.T.)

**Keywords:** idiopathic macular hole, estrogen, cone photoreceptor, mitochondria

## Abstract

The aim of this review was to identify a new potential explanation for the development of macular holes in relation to the female sex and to explain the possible underlying pathways. This approach was based on the evaluation of anatomical, physiological, and morphological analyses currently available in the literature. The findings showed that estrogen exerts a protective effect on the neuroretina and may influence Müller and cone cells. Both cell types are responsible for the building of the fovea structure. However, this protection may be lost due to the sudden decrease in estrogen levels during menopause. In conclusion, the fovea cones, through its sensitivity to estrogen and high energy consumption, may be very vulnerable to damage caused by a sudden changes in the concentration of estrogen in menopausal females. Such changes may result in cone degeneration, and thus a destroyed structure of the fovea, and may lead to the development of a hole in the fovea, as in the case of macular holes. This review revealed that under the decreasing influence of estrogen may cones play a key role with regard to the etiology of the development of macular holes. This aspect may be of strategic importance in prophylactic therapy for the prevention of the development of macular holes in premenopausal females or after ocular trauma.

## 1. Introduction

Idiopathic macular hole (IMH) is a common cause of impairment of vision in adults. The annual incidence of IMH has been shown to be 8.69 eyes per 100,000 individuals [1]. Although it primarily occurs in adults, it may also be diagnosed in children [2,3]. In previous studies, IMH was described as an age- and even more as a sex-related disorder. It should be noted that females have been linked to a three-fold-higher risk of developing IMH compared with males [1], and a higher incidence is especially observed in females with advanced age [4]. Therefore, understanding the role of female sex hormones is important in order to elucidate the etiology of this disease.

The retina originates from the central nervous system (CNS) and is already influenced by sex hormones in the fetal stage. It has been shown that the CNS of the female fetus is primed by high levels of testosterone [5]. Therefore, understanding the biochemical events occurring in the brain may assist in understanding retinal diseases, especially those related to gender. The majority of age-related changes in retinal gene expression are sexually divergent [6], and sex as well as age may influence the function and structure of the retina [7]. Moreover, the neuroprotective effect of estrogen on retinal cells has been repeatedly reported [8,9,10]. Although IMH has previously been discussed extensively in the literature, newly obtained evidence from research on the retina suggests a re-evaluation of the pathogenesis of IMH from a new therapeutic perspective. Driven by the high incidence of IMH in females, this review aims to identify new possible reasons for the development of this disease. It is possible that the etiology of IMH has an additional hormonal and neurodegenerative nature due to the biochemical effect of the estrogen hormone and the morphological anatomical structure of the fovea.

In general, previous studies have reported several systemic and ocular factors that are associated with the development of IMH.

There are systemic factors, like plasma fibrinogen, angiotensin-converting enzymes (ACEs), diabetes, and chymases, that can affect IMH development. High levels of fibrinogen have been significantly associated with a high risk of macula hole development. It has been proposed that a high level of fibrinogen may increase sensitivity to the power of vitreous traction and inhibit the blood supply of the macula [11]. Angiotensin-converting enzyme has been also associated with IMH [12]. A higher prevalence of diabetes has been found in patients without macular holes; therefore, diabetes seems to be protective against IMH [4]. However, this aspect remains unclear. High levels of chymases result in IMH. This influence could be explained with the inhibition of Müller cell proliferation and their atypical characteristics in the macula. Thus, under the exposure of chymase enzymes, the Müller cells can cause fibrosis and apoptosis [13]. The production and degeneration of collagen is also related to the influence of chymases [13,14].

Ocular factors that are associated with IMH development include glaucoma [11], myopia, shorter axial length, and vitreous changes. IMH has been associated with a shorter axial length of about 22.94 mm [15]. In addition, IMH can also occur at a young age, although it is associated with severe myopia in such cases [16]. The role of the vitreous during the development of IMH has been extensively studied. Eyes with macular holes demonstrated a significant initial phase of vitreous degeneration [17]. IMH has been thought to be caused by focal contraction of the vitreous in the fovea region [18], through tangential traction in liquefied pocket of the thin vitreous in the premacular area [19]. Another study suggested that dynamic vitreous traction is associated with ocular rotations due to perifoveal vitreous detachment from the pericentral retina. This mechanism led to foveal dehiscence and anterior contraction on the foveola, which ended in the IMH formation [20]. The hydration theory states that the development of IMH may be due to damage to the inner retinal layer, resulting in aggregation of the liquefied vitreous in the middle and outer layer of the retina [21]. Subsequent studies, using mathematical and physical models to determine the evolution of IMH formation, have reported that eyes with V-shaped vitreofoveal attachment were associated with early morphological alteration in the outer layers of the fovea, whereas the eyes with U-shaped attachment showed morphological changes in the inner layer of the retina. Both morphological and mechanical differences in vitreofoveal attachment result in the development of IMH [22].

Also, Müller glial cells have been thought to play a role in the pathogenesis of IMH. This could be explained by the induced migration of Müller glial cells through the complement pathway and α2 macroglobulin, which then contributes to epiretinal membrane formation in IMH [23]. Lastly, estrogen and the role of sudden changes in hormonal balance are involved in the development of IMH [4]. The latter will be discussed in more detail in this review.

## 2. Biochemistry

### 2.1. Retina and Estrogen

A number of studies observed the expression of estrogen receptor (OR) mRNA in the neuroretina, including the ganglion cell layer, the inner nuclear layer, and the outer portion of the outer nuclear layer of the retina [24]. It was also found that mRNA encodes different estrogen receptors, namely ORβ and ORα, and that both were present in the human ocular posterior segment. The difference has been located in the estrogen receptor; thus, ORβ protein was especially localized in the ganglion cell layer and the choroid [25]. Interestingly, the estrogen receptor alpha (Erα) protein was found in the retina as well as in the retinal pigment epithelium (RPE) of young female eyes, but not in eyes of males or post-menopausal females [26]. This difference in presence of Erα protein regarding sex and age requires further research and may assist previously unsolved approaches regarding eyes disease.

There are three important types of estrogen, namely estrone (E1), estradiol (E2), and estriol (E3) [27]. The levels of estradiol E2 have been suggested to be of considerable value in the preservation of healthy visual performance, especially in older females [27].

Estrogen is not only a female hormone; males also produce estradiol. Although, there is a difference in estradiol levels in aging between females and males. Hence, androgen production in males decreases with age, but no decrease in plasma estradiol has been described. This finding seems to be related to increased aromatase activity and fat mass as a result of aging in men [28].

### 2.2. Protective Effect of Estrogen in the Retina

#### 2.2.1. Energy Homeostasis

Estrogen influences the induction of various proteins which are localized in human retinas and other eye tissues, namely cytochrome P-450, heat shock protein 27, c–*fos*, c–*myc*, tumor growth factor α, pS2, α 2-macroglobulin, etc. This explains why estrogen plays a crucial role in important biological processes starting at the cellular level, such as development, and differentiation of cells [26,29]. Estrogen has been shown to be involved in the control of an enormous network of genes via estrogen-related receptors (ERRs; nuclear receptors). These regulatory gene networks are involved in important metabolisms including energy homeostasis from fat and glucose metabolism inclusive of mitochondrial biogenesis [30,31].

#### 2.2.2. Extracellular Matrix

Estrogen has also been involved in the expression of other important retinal genes such as the fibulin-3 gene, which belongs to secreted glycoproteins. In other words, estrogen can influence the extracellular matrix in the outer retina through the transformation of fibulin-3 [32].

#### 2.2.3. Antioxidant Influence

Estrogen also has a positive and protective antioxidative influence. The protective mechanism of βE2 occurs by means of antioxidative effects that are retraced through the activation of NRF2 via two pathways: a fast, non-genomic PI3K/AKT response, and a genomic-type ER-dependent response [33]. Estrogen and ERs can affect multiple processes via popular and well-documented pathways. Thereby, the ligand-bound ERs can take various routes, for example by targeting the estrogen-response elements (ERE) or the promoters of target genes directly. The ligand-bound ERs can even affect the transcription of genes without EREs; these can pass through interactions with other transcription factor complexes such as SP-1 (GC-rich SP-1 motifs) or Fos/Jun (AP-1-responsive elements) [34].

A study on rats showed that the application of βE2 induced the transportation of NRF2 from the cytoplasm to the nucleus, especially in the outer nuclear layer of the retina. As a consecutive reaction, NRF2 induces the expression of phase-2 antioxidant enzymes such as thioredoxins 1 and 2, superoxide dismutase 1 and 2, catalase, and glutaredoxins 1 and 2. The application of βE2 also caused a reduction as well as a production of reactive oxygen species (ROS), and in this way induced damage to the retina [33].

It has also been suggested that estrogen protects the RPE from oxidative stress. ARPE-19 cells, a human RPE cell line, expressed high levels of ERα and ERβ and 17-β estradiol (17β-E2), which protected ARPE-19 cells from oxidative stress via a ERβ-dependent process. It has been demonstrated that 17β-E2 induced cytoprotection by contributing to the maintenance of mitochondrial function, and additionally by the reduction in ROS production and induction of cellular antioxidant genes [35]. Therefore, it is not surprising that non-feminizing estrogens have been suggested as potentially useful compounds for the neuroprotection of retinal cells [8].

#### 2.2.4. Estrogen Treatment in Eye Disease

Several studies have linked estrogen to the therapeutic strategy of certain retinal diseases. Examples include the use of estrogen as prophylactic therapy for Leber’s hereditary optic neuropathy by targeting ERβ [36]. It was also suggested as therapy for glaucoma, since it reduces retinal ganglion cell loss induced by high intraocular pressure in rats [37,38]. Estrogen reduced the visual system damage and rescued photoreceptor cells after ocular blasts in mice [39]. In addition, it has been considered as a therapy for proliferative vitreoretinopathy and other proliferative retinal diseases. Thereby, it appears to inhibit TGF-β2-induced collagen contraction mediated by RPE cells by inhibiting the expression of the mesenchymal markers α-SMA and fibronectin, interleukin-6 release, and TGF-β2-induced Smad2 and MLC phosphorylation [40]. Moreover, in diabetic retinopathy, it has been demonstrated that 17β-estradiol (E2) exerts protective effects on retinal ganglion cells in a high-glucose environment [41].

### 2.3. Influence of Estrogen on Mitochondria and Cone Cells of the Retina

Few previous studies have investigated the possible role of estrogen in cone cells. Identification of the genes expressed in cone photoreceptors has revealed that one of the isolated genes encoded ZBED4, a novel protein localized in cone photoreceptors and glial Müller cells in the human retina. Several putative ZBED4-interacting proteins have been identified, including a co-repressor of the ORα [42]. A similar finding has been demonstrated in the cone cells of zebra fish. It was noted that ES1—a novel mitochondria-enlarging factor localized in cone mitochondria—contributes to the formation of mega-mitochondria in the cone cells. Knockdown of ES1 markedly reduced mitochondrial size in cone cells. Therefore, it has been suggested that ES1 supports energy production in the zebra fish retina via mitochondrial enlargement [43]. Theoretically, similar findings in the human retina would imply that estrogen may exert a direct effect on both types of cells (i.e., cone and Müller glial cells). Moreover, this finding suggests that cone cells are, to a certain extent, dependent on estrogen levels for their energy production. Hence, sudden changes in the estrogen levels, such as those occurring during menopause, may exert harmful effects on cone cells, leading to their degeneration.

### 2.4. Role of Estrogen in Idiopathic Macula Hole

There are various findings related to the effect of estrogen in the development of IMH. Overall, a positive effect of estrogen against IMH has been reported in the literature [11]. Indications for positive effects of estrogen against the pathogenesis of IMH were identified in RPE cells, where the female sex hormones 17β-estradiol and progesterone inhibited TGF-β2-induced collagen contractions [40]. Another effect discovered was the induced estrogen inhibition of collagen gel contraction by glial cells [44]. Controversially, some publications also reported the development of macular holes under estrogen treatment [45,46]. The first report evaluated the levels of estrogen in the vitreous body of patients with IMH and observed that vitreous E2 levels were higher than E1 levels. This result was attributed to the conversion to E2 from E1 by 17-β hydroxy-dehydrogenase, and to the local production of estrogen based on the expression of P450 aromatase in the human eye. Other possibilities mentioned in the literature include the effect of pro-inflammatory cytokines promoting the aromatization of testosterone to E2 and glucuronidation altering E2 and E1 [16]. Another study examined the possibility of neurosteroid production in the retina. They compared the levels of steroid hormone E2 and testosterone in the vitreous and in the serum of patients with vitreoretinal diseases (i.e., idiopathic epiretinal membrane, IMH, proliferative diabetic retinopathy, and rhegmatogenous retinal detachment). In all these vitreoretinal diseases, females had significantly higher E2 levels in the vitreous than in the serum. In contrast, males had significantly higher levels in the serum than in the vitreous body. Therefore, it has been suggested that the synthesis of E2 is locally increased only in female eyes. The investigators proposed differences between males and females in the quantity of aromatase produced in the brain and retina. They concluded that this high vitreous E2 level in females is increased due to the production of aromatase activated by reactive astrocytes because of the damage caused by these diseases. The production of E2 by astrocytes and Müller cells may be responsible for the suspected local production of E2 [47]. In conclusion, the elevation in vitreous estrogen levels should be considered separately from the development of IMH through decreased systemic estrogen levels as observed during menopause, after hysterectomy, or after systemic anti-estrogen treatment. However, the protective effect of estrogen against the development of IMH is often reported. This knowledge may be confirmed through anti-estrogen treatment. It should be mentioned that some studies have reported a relationship between anti-estrogen treatment (e.g., tamoxifen) and the development of IMH [48,49,50]. However, the exact mechanism involved in this process remains unknown. The effect of tamoxifen on Müller cells has already been studied. Müller glial cells influence other retinal cells and play important roles in the retina, including reprogramming, regeneration, and restoration of vision [51]. On the other hand, an influence on RPE cells has also been reported. Thus, it has been found that treatment with tamoxifen and toremifene may result in retinal defects. This occurs via a dose-dependent decrease in the glutamate uptake of RPE cells, which has been noted in porcine RPE cells as well as in human RPE cell lines [52]. Also, it has been proposed that the toxicity of both photoreceptor and RPE cells caused by anti-estrogen drugs may be attributed to the development of anti-estrogen maculopathy [53].

## 3. Anatomical and Morphological Effects of Estrogen on the Fovea

### 3.1. Müller Glia and Cone Cells in the Theory of Gass

The structure of the fovea regarding Müller glia and cone cells was first described by Yamada [54]. This finding was subsequently used by Gass in forming his hypotheses on IMH formation, based on the finding that the inner half of the fovea is composed of an inverted cone-shaped zone containing Müller cells (Müller cell cones). The more interesting hypothesis suggested that Müller cell cones bind the receptor cells in the fovea. Thus, the Müller cell cone is the primary structural support for the fovea. Gass presumed that an absence of this plug of glial cells results in the formation of a macular hole [55]. An anatomical association between cones and IMH was also reported by Ezra [56]. He suggested that the variation in cone photoreceptor density in the operculum (40–50% contain photoreceptors) probably reflects the amount of foveal tissue avulsed during the formation of the hole. He corroborated his hypothesis through previously obtained clinicopathological findings, showing a lower anatomical success rate following surgery in the operculum [56].

### 3.2. Morphological Changes in the Retina Induced via Treatment with Tamoxifen

The morphological changes in the retina following a treatment with tamoxifen, a selective OR inhibitor, include foveal cystic changes with an outer retinal defect determined through optical coherence tomography (OCT) [48,57]. Also, macular pigment absorption and incorporation in retinal tissue after longer tamoxifen use has been suggested [58]. Gualino et al. used OCT analysis to reveal a foveolar cystoid space with focal disruption of the photoreceptor line. However, there was no evidence of macular oedema or thickening [59]. This morphological finding due to tamoxifen treatment may be related to the direct effect of tamoxifen on the OR in the fovea cone, which is probably responsible for the disruption of the photoreceptors. This hypothesis may be confirmed using ES1, a novel mitochondria-enlarging factor localized in the cone mitochondria [43]. The influence of menopause, hence, a lack of estrogen, on the retina may be similar to that observed following treatment with tamoxifen. Furthermore, a thinning in the parafoveal regions has been observed in females compared with males [60]. In addition, the incidence of IMH in females has been reported to increase with age. This may be simply attributed to the anatomical changes observed in the retina due to ageing. It should be noted that a morphological examination of the retina using OCT showed that the inner retinal thickness decreased by 0.5 μm per year and was 6.1 μm thinner in females compared with males [61]. Therefore, considering the important role of Müller glial cells, the natural architecture of the macula showed that the inner layer of the fovea is formed by Müller cell cones [54], taking into consideration the previously reported effects of estrogen on retinal cells. All these factors together may explain the development of IMH and not elsewhere in the retina following treatment with tamoxifen or lack of estrogen.

## 4. Functional Examination

### 4.1. Postive Effect of Estrogen on Vision

It has been proposed that hormonal changes may be linked to alterations in retinal function [62]. Studies have shown differences between the retinas of males and females. Thus, it has been determined that the young female retina has a better function than the male one, regarding electroretinogram (ERG) measurements. Using multifocal ERG, it was demonstrated that implicit time (IT) results vary inversely with the presumptive levels of estradiol in both sexes. Shorter ITs were noted in younger females compared with males, whereas similar ITs were detected in older females and males, and the longest ITs were seen in females with hysterectomies. The neuroretinal function in older females was clearly worse than that in younger females. In contrast, neuroretinal function did not differ between younger and older males in all IT evaluations. A possible explanation for these results is the involvement of age-related changes in the average estradiol levels [63]. The positive effect of estrogen on the retina has been reported by Chaychi et al. Through ERG, they showed that sex and age may influence retinal function and structure in rats. Furthermore, the ERG of premenopausal female rats was compared with that observed in menopausal female rats as well as male rats. They demonstrated that cycled female rats had better retinal function compared with menopausal female rats and suggested the beneficial effects of the estrus cycle on retinal function [7]. A similar investigation was found, as pattern reversal evoked potentials (PRVEPs) examination has been recorded in females throughout four different phases of the menstrual cycle. The results showed that the PRVEP latency recorded during the ovulatory phase was statistically significantly shorter than that of other phases. The significant decrease in PRVEP latencies was attributed to the effect of estrogen on neural transmission of the visual pathways [64].

### 4.2. The Role of the Cone Cells in the Development of Idiopathic Macula Holes

Cone cells are high-energy-consuming cells; they use more ATPs for the activation of transducin and rhodopsin phosphorylation than rods [65]. To the best of our knowledge, there are, to date, only two studies that examined the function of cone cells in the development of IMH. 

The first study was conducted by Andréasson et al. The preoperative retinal function was measured using full-field ERG, and multifocal ERG was correlated to postoperative visual acuity after surgery for the treatment of IMH. It was observed that cone IT in the full-field 30 Hz flicker ERG, reflecting retinal function, was significantly prolonged prior to surgery compared with that of aged-matched controls. Thus, cone IT in full-field 30 Hz flicker ERG has been suggested as a predictor of visual outcome after surgery for the treatment of IMH [66]. 

The second study was performed by Ştefănescu-Dima et al. The aim of this study was to highlight the anatomical and functional changes in early stages of posterior vitreous detachment (PVD), using OCT and full-field ERG, and allowing for timely and correct treatment. The response of cone cells determined through full-field ERG was also proposed as a marker of retinal damage in macular pathology due to PVD. Moreover, it may be used to evaluate retinal function in macular pathology [67]. Furthermore, a recent study demonstrated that the diameter of inner/outer segment and external limiting membrane defects are among the most important factors affecting best-corrected visual acuity in IMH [68].

## 5. New Epidemic Study

**Figure 1:**
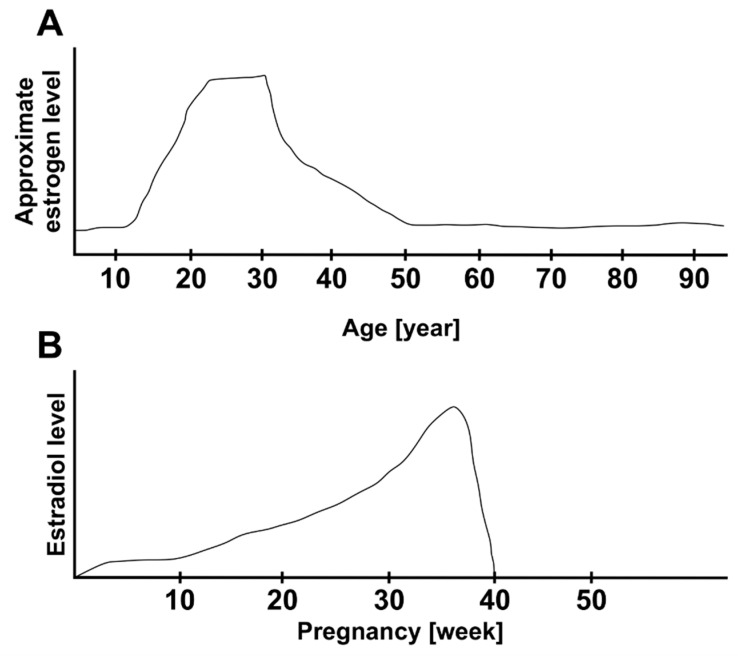
Overview of estrogen level change during lifetime (**A**) and during pregnancy (**B**)**.** Adapted from [70,71].

A new epidemic cohort study examined risk factors for developing IMH among a large population of approximately 4,500,000 individuals. According to the study, IMH occurred earlier in woman than man. Also, postmenopausal women who had given birth to two or more children showed a greater risk of IMH than those who had not been pregnant (hazard ratio: 1.8) [69]. This is a very interesting result. It is known that hormonal changes regarding the estrogen level occur during pregnancy. Estrogen concentration increases constantly during pregnancy, achieving a peak, then it decreases suddenly at the end of the pregnancy. Figure 1 gives an overview over the change in estrogen level during lifetime (A) and during pregnancy (B) [70,71]. This increase in estrogen level during pregnancy and abrupt decrease at the end may have a crucial effect on the inverted cone-shaped zone of Müller cells. This might, on the one hand, induce the first weakening of the fovea architecture and, on the other hand, affect the vitreous cortex. At a young age, these proposed effects of estrogen may be compensated after a normalization of estrogen level post pregnancy, and the changes in fovea are reversible. A repeated and rapid but irreversible decrease in estrogen levels as it occurs during menopause may irreversibly weaken the cohesion of Müller cell cones and finally destroy the plug of glial cells, which results in macular holes. This upregulation and then rapid downregulation of the estrogen level during pregnancy may have an intense effect on the fovea. This might explain the association between childbirth and an increasing risk of IMH development. Another very interesting point, which has been reported in the epidemic study of Hwang et al., is that the history of postmenopausal replacement hormone therapy (HRT) was not associated with the risk of IMH development. Therefore, the authors concluded that this finding dost not support the estrogen theory [69]. We assume that the rapid change in estrogen levels is more relevant than a possible late exogen estrogen treatment, which probably occurs later in the postmenopausal period. Postmenopause may be noticed late: it is often diagnosed in retrospect, usually due to the absence of menstruation. In this non-diagnosable time until the supply of HRT, the sudden drop in estrogen might already be setting in and the crucial changes in cone cells and Müller cells occur, leading to IMH development. 

Another important point, which may confirm the relevance of estrogen level continuity, is the estradiol level in men. The absence of a rapid decrease in estradiol level in aging males compared with females can also be a plausible observation, where elderly females suffer from IMH more than elderly males. This assumption can be consistently proven without the rapid decrease in the effect of positive estradiol on foveal architecture and function in the retinas of aging males.

## 6. Discussion

As mentioned, different studies have tried to explain the influence of estrogen in IMH development in various ways. There are a few experimental studies, including histologic and biochemic evaluations, and some epidemic studies. A summary of relevant studies is emphasized in Table 1. Despite the differing results and the sometimes-small study size, the majority of the studies point in the same direction as the recent epidemic study by Sunsoon Hwang et al [69]. However, all these studies are unable to explain the particular molecular and biochemical mechanisms underlying IMH development, especially regarding the cone and Müller cells. 

Retrospectively, Gass built a gold standard in regard to explaining IMH formation with his hypothesis [55]. At this time, he was unable to confirm his hypothesis at a molecular level. Since Gass’s work, enormous technological advantages in retina imaging, functional measurements, and biochemical as well as molecular evaluations have occurred. 

This review connected the hypothesis of Gass and the molecular and the hormonal effect of estrogen on cone and Müller glial cells. It is remarkable that Gass came up with this great hypothesis, even without detailed knowledge about molecular events in cone cells and Müller glial cells under the influence of estrogen. Due to all these findings regarding the positive effect of estrogen on IMH development, we assume IMH has a neurodegenerative nature. How to use this positive effect of estrogen against IMH still needs to be discussed. However, a systemic supply of estrogen can provide undesirable side effects, for example, a high risk of systemic thrombosis, tumor development, etc. The creation of an estrogen receptor agonist, which could be specific to cone cells, but without systemic adverse events, could be an interesting approach. A local supply of estrogen eye drops may be revolutionary for future treatment. On the other hand, it is very important to identify the source of vitreous estrogen. Perhaps it is possible to make a specific programming and intelligent activation of vitreous Astrocyte targeting local estrogen production in the fovea area. This idea needs more research in the future.

## 7. Summary

**Figure 2:**
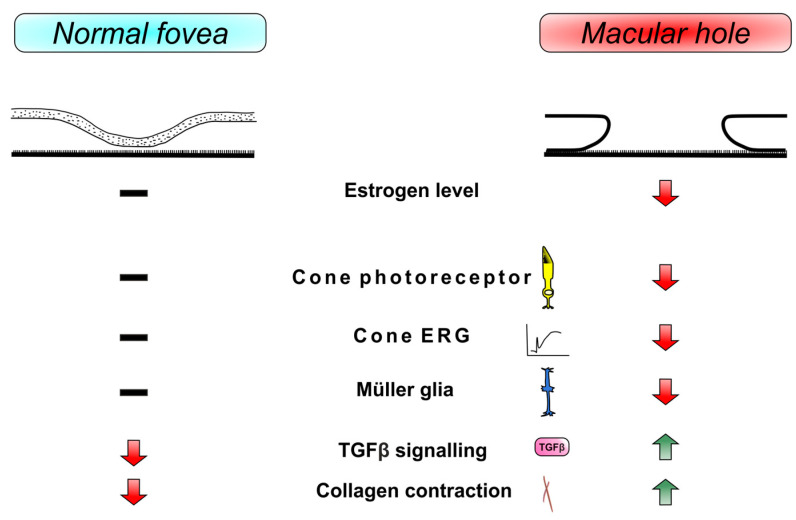
Overview of normal fovea and macular hole formation.

Gass reported on the opportunity of the Müller cell cone to bind to the fovea structure [55]. It is possible that morphological changes in the fovea, such as those observed in IMH, are partially related to changes in cone cells. And there is an abundant presence of cone cells in the fovea region. As previously mentioned, estrogen influences Müller glia and cone cells [42,43]. Currently, our understanding of the precise effects of estrogen on cone cells is limited. The hormonal effects of estrogen and its biochemical involvement in the neuroretina are very complex. Therefore, even a daily regulation of ERRβ, a transcriptional regulator of energy metabolism protecting rod photoreceptors from dystrophy, in the photoreceptor cells of rats has contributed to their adaptation to 24 h changes in metabolic demands [72]. It is possible that fovea cones, as hard-working cells with high rates of consumed energy [65] through their sensitivity to estrogen, which, in turn, is important for cell protection and is involved in energy metabolism [30,31,33], react to degeneration caused by the sudden decrease in estrogen level during menopause, which results in macular holes. This interpretation may be confirmed by the objectifiable measurement of the function of cone cells in patients with IMH. Another important finding is the difference between the systemic and local vitreous estrogen levels in IMH. However, this finding remains controversial with limited available evidence currently. Therefore, future studies focusing on vitreous estrogen levels in patients with retinal disease are needed. A summary illustration in Figure 2 shows a basic view of players mentioned in the development of IMH regarding estrogen influence.

## 8. Conclusions

Understanding the effect of hormones is essential to better comprehend vitreoretinal disease and determine the most appropriate treatment. However, this research area is at an early stage. Fovea cone energy production most likely depends on estrogen. Due to the presence of OR in cone mitochondria, it may be possible that the fovea cones, through their sensibility to estrogen and high rates of energy consumption, are very vulnerable to damage through a sudden change in the systemic levels of estrogen in females. This may be key in improving current strategies for the management of IMH. Finally, strategies aimed at exploiting the effects of estrogen on cone cells may play an important role in prophylactic therapy for IMH in the future.

## Figures and Tables

**Table 1:** Summary of relevant studies.

Study	Species	Relevant Results	Category
Zhu C et al., 2015	Rats	Protective mechanism of βE2 through antioxidative effects through NRF2 activation	Positive effect of estrogen on retina
Giddabasappa A et al., 2010	Human	17β-E2-mediated cytoprotection occurred through the preservation of mitochondrial function, reduction in ROS production, induction of cellular antioxidant genes	
Honig MG et al., 2021	Mice	Estrogen reduced the visual system damage and rescued photoreceptor cells after ocular blast in mice	
Farber DB et al., 2010	Human	ZBED4, a novel protein localized in cone cell and glial Müller cells, several putative ZBED4-interacting proteins including a co-repressor of the ORα	
Masuda T et al., 2016	Zebra fish	ES1, a novel mitochondria-enlarging factor localized in cone mitochondria, contributes to the formation of mega-mitochondria in cone cells	
The Eye Disease Case-Control Study Group, 1994	Human	Positive effect of estrogen against IMH	Epidemic study
Sungsoon H et al., 2022	Human	IMH occurs earlier in woman than men, postmenopausal women with two or more children showed a greater risk for IMH than those who had not been pregnant	
Kimura K et al., 2014	Human	Positive effect of estrogen on RPE cells, estrogen appears to inhibit TGF-β2-induced collagen contraction	Collagen contraction study
Qiu QH et al., 2012	Human	Estrogen can inhibit collagen gel contraction, which is caused by cultured human retinal glial cells	
James M et al., 1980	Human	Development of macular holes under estrogen treatment	Controverse study
McDonnell PJ et al., 1982	Human	Development of macular holes under estrogen treatment	
Inokuchi N et al., 2015	Human	Vitreous E2 level is higher than the E1 level in IMH patients	
Nishikawa Y et al., 2017	Human	Production of E2 by astrocytes and Müller cells may be responsible for the suspected local production of E2	
Chung SE et al., 2010	Human	Relationship between antiestrogen treatment and IMH development	Antiestrogen study
Cronin BG et al., 2005	Human	Relationship between antiestrogen treatment and IMH development	
Torrell Belzach N et al., 2020	Human	Relationship between antiestrogen treatment and IMH development	
Mäenpää H et al., 2002	Human	Tamoxifen: dose-dependent decrease in the glutamate uptake of RPE cells, which has been noted in porcine RPE cells as well as in human RPE cell lines	
Yamada E et al., 1969	Human	Structure of fovea in regard to Müller glia and cone cells	Fovea structure
Gass JD, 1999	Human	Important hypothesis, suggesting that Müller cell cones bind the receptor cells in the fovea together	
Ezra E, 2001	Human	Anatomical association between cones and IMH	
Yilmaz H et al., 1998	Human	Pattern reversal evoked potentials (PRVEP) latency during the ovulatory phase was significantly shorter than that of other phases	Retina function
Andréasson S et al., 2014	Human	Cone IT in full-field 30 Hz flicker ERG has been suggested as a predictor of visual outcome after surgery for IMH	
Ştefănescu-Dima AŞ, 2016	Human	Response of cone cells determined through full-field ERG was proposed as a marker of retinal damage in macular pathology	

## Data Availability

The datasets generated during and/or analyzed during the current study are available from the corresponding author on reasonable request.
